# Bone Morphogenetic Protein-, Antimicrobial Agent-, and Analgesic-Incorporated Nanofibrous Scaffolds for the Therapy of Alveolar Clefts

**DOI:** 10.3390/pharmaceutics14020374

**Published:** 2022-02-08

**Authors:** Pang-Yun Chou, Demei Lee, Chi-Chang Weng, Ren-Chin Wu, Chien-Tun Liao, Shih-Jung Liu

**Affiliations:** 1 Department of Mechanical Engineering, Chang Gung University, 259 Wen-Hwa 1st Road, Taoyuan 33302, Taiwan; chou.asapulu@gmail.com (P.-Y.C.); dmlee@mail.cgu.edu.tw (D.L.); jordan860814@gmail.com (C.-T.L.); 2 Craniofacial Research Center, Department of Plastic and Reconstructive Surgery, Chang Gung Memorial Hospital at Linkou, Taoyuan 33305, Taiwan; 3 Department of Medical Imaging and Radiological Sciences, Chang Gung University, Taoyuan 33302, Taiwan; ccweng@mail.cgu.edu.tw; 4 Department of Pathology, Chang Gung Memorial Hospital and Chang Gung University College of Medicine at Linkou, Taoyuan 33305, Taiwan; renchin.wu@gmail.com; 5 Bone and Joint Research Center, Department of Orthopedic Surgery, Chang Gung Memorial Hospital at Linkou, Taoyuan 33305, Taiwan

**Keywords:** alveolar cleft, nanofibrous scaffold, bone morphogenetic protein, antimicrobial agent, analgesic

## Abstract

An alveolar cleft is a bone defect in the maxillary arch. Although the use of autologous iliac bone grafts to repair alveolar clefts is the preferred treatment method, donor-site morbidity remains a concern. In this study, we incorporated bone morphogenetic protein (BMP), an antimicrobial agent, and an analgesic into nanofibrous scaffolds for alveolar cleft therapy. Three-dimensional (3D) printing and coaxial electrospinning techniques were used to fabricate the scaffolds. BMP-2, ketorolac, and amoxicillin were used as the growth factor, analgesic, and antimicrobial agent, respectively. The in vitro properties of the nanofibrous scaffolds were characterized, and in vivo efficacy was evaluated in a rat alveolar-cleft model. The empirical data indicated that the biomolecule-incorporated scaffolds offered extended discharge of BMP-2, amoxicillin, and ketorolac for >4 weeks. The animal test outcomes also demonstrated favorable bone healing at the cleft site. Biomolecule- and drug-incorporated nanofibrous scaffolds demonstrated their efficacy in alveolar cleft treatment.

## 1. Introduction

An alveolar cleft is a congenital craniofacial defect connecting the oral and nasal cavities. The reconstruction of alveolar clefts demands closure of the oronasal fistula, bone graft implantation for the maxillary deficit, and the restoration of the alignment of the upper gingival and palatal mucosa [1]. The optimal time for alveolar cleft repair is at primary school age, when the patient’s permanent teeth are beginning to erupt [2]. In the literature, autologous iliac bone graft remains the most practical material to facilitate maxillary bone development for tooth eruption [3,4]. However, for patients with failed bone grafting or patients with a bilateral alveolar cleft, the alternative treatment wherein optimal bone grafting material is used for alveolar cleft reconstruction should be considered when the volume of cancellous bone grafts is limited or not adequate for the procedure.

In tissue engineering, functional biomaterials are developed to restore, maintain, improve, or replace defective organs and tissues [5]. In addition to enabling structural imitation to improve recovery and regeneration, tissue engineering requires the integration of biomaterials, biochemical, and physicochemical factors to implement ideal scaffolds [6,7]. To reduce donor-site morbidity, various strategies have been investigated, including the adoption of allogeneic bone and the use of artificial substitutes [8], bone morphogenetic proteins, and growth factors [9,10,11,12,13].

Surgical infections are not uncommon, and can result in postsurgical pain and impeded healing. The development of such infections may require prolonged hospitalization, the extended use of antibiotics, increased medical costs, and even additional surgical intervention [14]. Studies have reported that, depending on the type of surgery, infections may develop in 5–20% of surgeries, resulting in 7–11 additional days of hospitalization and a 2–11 times higher probability of mortality [15]. Additionally, postoperative pain remains a major concern. Despite advances in pain management, some patients still experience postoperative pain. Postoperative pain can influence the patient’s surgical outcomes; the patient’s level of comfort and satisfaction with clinic management; and the subsequent evolution of tachycardia, hyperventilation, reduction in alveolar ventilation, transition to chronic pain, deficient wound recovery, and insomnia [16,17].

In this study, we developed degradable polylactide (PLA) scaffolds and drug-eluting poly(lactide-co-glycolide) (PLGA) nanofibers for the treatment of alveolar bone defects through the use of 3D printing [18,19] and coaxial electrospinning technologies. The scaffolds used for bone repair must be bioactive, biocompatible, and biodegradable and must possess appropriate mechanical strength. PLA is a degradable polymeric material with superior properties that has been extensively researched. The material has a glass transition temperature of 60–65 °C, a melting temperature of 130–180 °C, and an elastic modulus of 2.7–16 GPa. PLA has been demonstrated to be an excellent material for various healthcare applications, including tissue engineering, regenerative medicine, the fabrication of cardiovascular stents, and orthopedic interventions [20], owing greatly to the polymer’s favorable biocompatibility and to its safe degradation products [21]. The 3D printing of polymeric materials is a novel technique and solves complex medical problems in bioengineering and biomedical research. This versatile technology has been expanding rapidly due to the increased demand for customized and personalized medical products, pharmaceuticals, and other equipment [22]. The technology has also become the gold standard for constructing scaffolds for tissue engineering [23].

In addition, we used bone morphogenetic protein (BMP), antimicrobial agents, and analgesics incorporated nanofibers for drug delivery in the alveolar cleft reconstruction procedure. Bone proliferation and regeneration is an extended procedure that includes the sequential expression of growth regulatory factors by osteoblasts as they grow and eventually differentiate. BMP-2 promotes the expression of other BMP genes in the bone cell differentiation process; furthermore, through paracrine signaling with other BMPs, BMP-2 prompts cell differentiation and bone formation during the remodeling process [24]. Ketorolac belongs to the nonsteroidal anti-inflammatory drug (NSAID) class and is an FDA-approved medication used for the management of acute moderate to severe pain [25] and is commonly used for postoperative pain management. Amoxicillin, a moderate-spectrum, bacteriolytic, β-lactam antimicrobial agent in the aminopenicillin family, is used to cure susceptible Gram-positive and Gram-negative bacteria [26] and has been used to treat bacterial infections, including tonsillitis, bronchitis, pneumonia, and infections of the ear, nose, throat, skin, and urinary tract. PLGA is a polymeric biomaterial widely employed for controlled drug release for its excellent biocompatibility and biodegradability and for its capacity to deliver various small-molecule drugs, peptides, and proteins [27,28]. Electrospinning, moreover, is a versatile process for making uniform and ultrafine nanofibers [29] that mimic the nanofibrous extracellular matrix of human body tissues and accelerate tissue regeneration. Nanofibrous membranes can also be tailored to provide the sustainable release of biomolecules and drugs [30]. These unique features grant nanofibers with powerful capabilities for various applications in tissue engineering and drug delivery.

After fabrication, the in vitro release patterns of the drugs and growth factors from the nanofibrous scaffolds were assessed; the release-pattern and growth-factor properties were estimated through high-performance liquid chromatography (HPLC) and enzyme-linked immunosorbent assays (ELISA), respectively. Moreover, in vivo efficacy was assessed on a rat alveolar-cleft model through histological examination.

## 2. Materials and Methods

### 2.1. Manufacture of Growth-Factor- and Drug-Loaded Scaffolds

The polymeric materials employed were 50:50 PLGA (RG503, Mw = 24,000–38,000) and 75:25 PLGA (RG756, Mw = 7600–115,000). The biomolecules included BMP-2, ketorolac, and amoxicillin. The solvent used was 1,1,1,3,3,3-hexafluoro-2-propanol (HFIP; Sigma-Aldrich, St. Louis, MO, USA). All materials were provided by Sigma-Aldrich (St. Louis, MO, USA).

**Figure 1:**
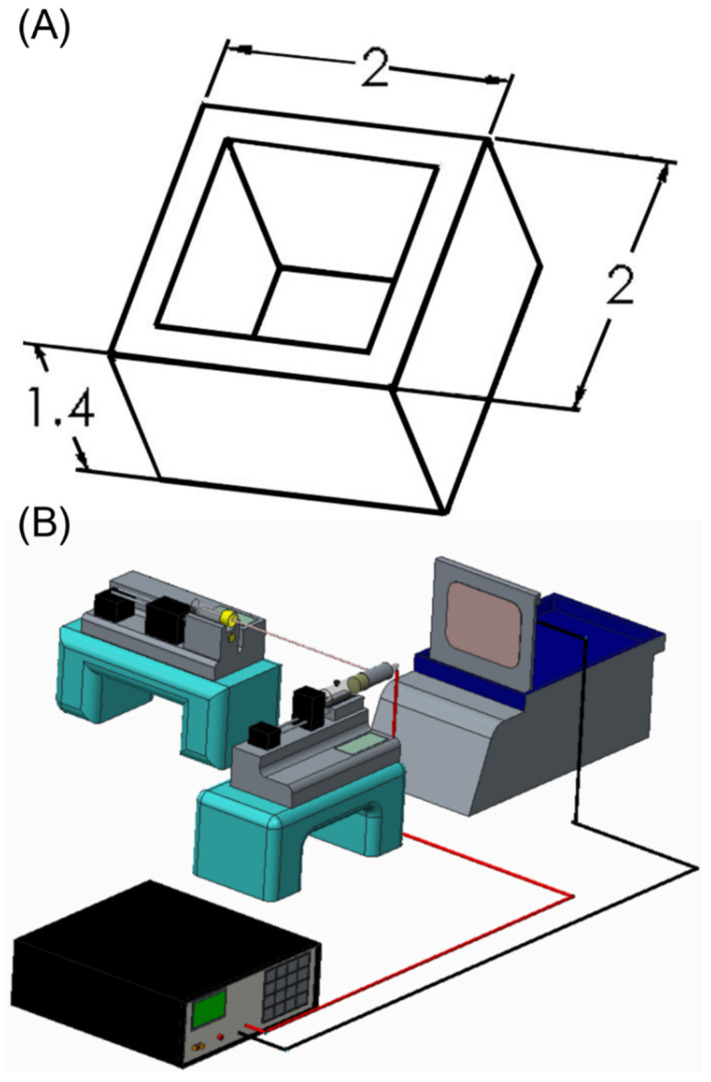
(**A**) Layout and dimensions of PLA scaffold (unit: mm, thickness = 0.5 mm), (**B**) the coaxial electrospinning device used to prepare the drug-eluting nanofibers.

Bioresorbable scaffolds were fabricated through a 3D printing process. The dimensions and layout of the scaffold are displayed in Figure 1A. To print the scaffolds, PLA filaments (Prolink Microsystems Corp., Taipei, Taiwan) and a fused deposition modeling printer (V2-B Dual Extruder Printer, Kraftmaker, Taipei, Taiwan) were used.

Bi-layered sheath–core-structured nanofibers, including drug- and growth-factor-loaded layers were prepared using a lab-scale coaxial electrospinning device (Figure 1B). To fabricate the drug-loaded layer, 50:50 PLGA (1,344 mg) and ketorolac (336 mg) were blended with 6 mL of HFIP as the mixture for the sheath layer. A combination of 50:50 PLGA (672 mg) and amoxicillin (168 mg) were blended with 3 mL of HFIP for the core layer. The mixtures were then transferred to two separate syringes and needles for coaxial electrospinning. The lumen sizes of the needles for the sheath and core layers were 1.20 and 0.43 mm, respectively. The solution transfer speed for the sheath layer was 0.9 mL/h, and the delivery speed for the solution at the core was 0.3 mL/h. Voltage was maintained at 18 kV, and the distance between the needle and the collection plate was 15 cm. The fabrication of the BMP-2-loaded layer proceeded according to the same process, except that the polymer used for the sheath layer was 75:25 PLGA (840 mg) mixed with 3 mL of HFIP, and the fluid at the core was a mixture of 20 μg of BMP-2 blended with 1 mL of phosphate buffered saline and 1 mL of bovine serum albumin.

After co-electrospinning, approximately 200 μm BMP-2- and drug-loaded nanofibrous membranes were obtained. The membrane was then inserted into the core of the 3D printed cage and the drug-eluting scaffold was complete.

### 2.2. Scanning Electron Microscopy

Electrospun drug- and biomolecule-loaded nanofibers were evaluated through scanning electron microscopy (SEM), and the nanofiber diameter distribution was determined using the results of 100 randomly selected fibers (*n* = 3) from the SEM micro-images through the use of the ImageJ code (National Institutes of Health, Bethesda, MD, USA).

### 2.3. Identification of the Sheath–Core Structure

The sheath–core structure of nanofibers was observed under a transmission electron microscopy (TEM; Jeol JEM-2000EXII, Tokyo, Japan). In addition, a laser-scanning confocal microscope (TS SP8X, Leica, Tokyo, Japan) was used to identify the presence of proteins in the electrospun nanofibrous mats. PLGA was used for the sheath layer, and recombinant enhanced green fluorescent protein (reGFP) was used for the material comprising the structure’s core. The nanofibers were examined at 487 nm.

### 2.4. Differential Scanning Calorimetry (DSC)

A differential scanning calorimeter (DSC-25, TA Instruments, New Castle, DE, USA) was used to evaluate the thermal behaviors of virgin PLGA, ketorolac, amoxicillin, and ketorolac- and amoxicillin-loaded PLGA nanofibers. The scanning temperature ranged between 30 and 250 °C, and the sample heating rate was 10 °C/min.

### 2.5. Wetting Angle Measurement

The wetting angles of the virgin PLGA, drug- and BMP-2-loaded nanofibrous membranes were evaluated. Distilled water was applied gradually to the surface of the nanofibers (10 mm × 10 mm) and monitored through a video camera (*n =* 3).

### 2.6. In Vitro Drug and Biomolecule Release

The release profiles of the drugs and growth factors from the electrospun nanofibrous membrane were investigated through an in vitro elution method. Nanofibrous specimens were placed in an assay tube (*n* = 3) holding 1 mL of phosphate buffered saline (PBS). The tube was incubated at 37 °C for 1 day, and the mixture was collected for assay. Subsequently, 1 mL of fresh PBS was added to the tube, and the procedure was replicated for 30 days. The drug concentration in the collected mixture was subsequently analyzed through HPLC [31,32], whereas the levels of BMP-2 were determined through ELISA.

### 2.7. In vivo Animal Test

Twenty S.D. rats (weighing approximately 300 g each) were used in the study and cared for in accordance with the regulations of the Department of Health and Welfare, Taiwan. All experimental protocols were approved by the Institutional Animal Care and Use Committee of Chang Gung Memorial Hospital (IACUC Approval No.: CGMH2018121905).

**Figure 2:**
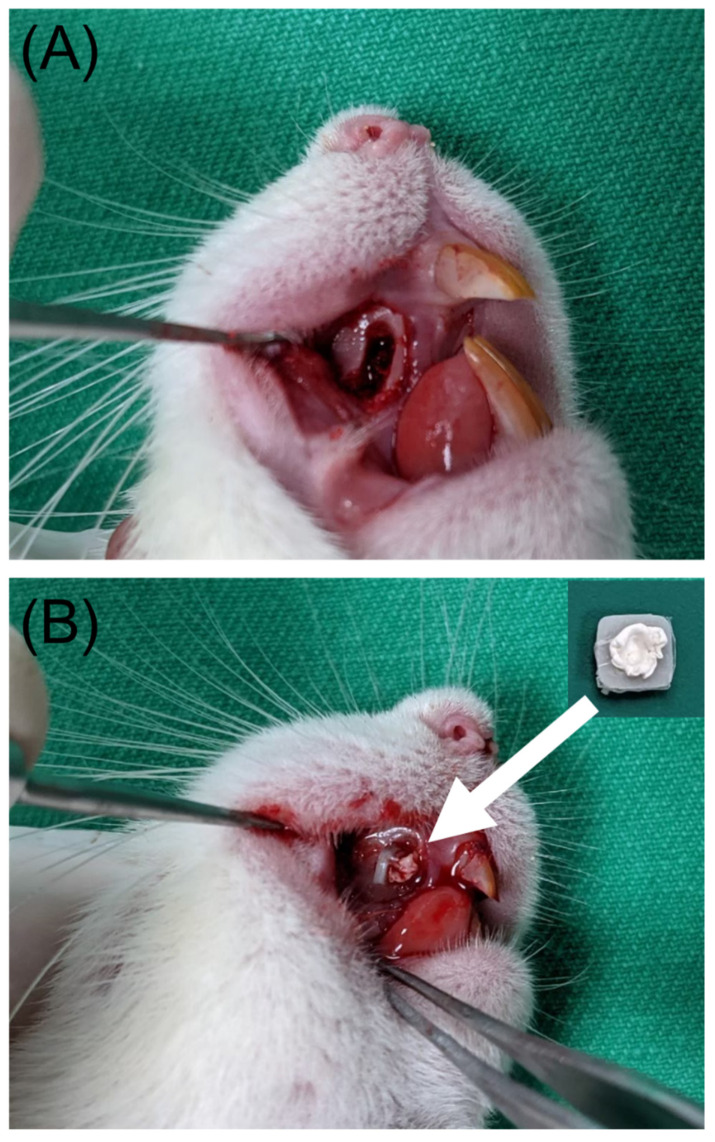
(**A**) Creating a bone cavity at the alveolar bone, (**B**) implantation of the drug- and biomolecule-incorporated nanofibrous scaffold in the bone cavity.

As illustrated in Figure 2, the rats were preliminarily anesthetized with isoflurane. A 1-cm incision was made in the right upper gingival site of each animal. This procedure was followed by using a 1.5-mm-diameter burr to create a cavity (2 mm × 4 mm × 2 mm) over the right upper alveolar area. The drug- and biomolecule-loaded nanofibrous scaffold was then implanted into the cavities of five of the rats. The wound was then closed with 3-0 Vicryl sutures. Tissues near the implantation site were sampled at 7, 14, and 28 days post surgery, and the drug levels in the samples were assessed through HPLC assays. Two rats in the drug/BMP-2 group were euthanized for histological examination, one at 7 days after surgery and a second at 14 days after surgery.

### 2.8. Postoperative Activity

The remaining 15 rats were used in the postoperative activity experiments and were separated into three groups of five (*n* = 5). The rats in group A underwent a surgical procedure to implant bare scaffolds (with no nanofibers) as a control group. The rats in group B underwent the same surgical procedure but were implanted with scaffolds loaded with ketorolac-, amoxicillin-, and BMP-2-eluting nanofibers. Comprising the normal group, the rats in group C did not undergo surgery or receive any implantation devices. The postoperative activities of each animal in each group were assessed through the use of an activity cage [33] that was equipped with nine sensors on its top section. The sensors were used to record the migration of a rat within the cage. When a rat shifted from one region of the cage to another, the sensor in the region the rat approached was triggered. The activity of each rat was monitored for 7 days by a computer equipped with an acquisition interface.

### 2.9. Micro-CT Examination and Bone Growth Evaluation

**Figure 3:**
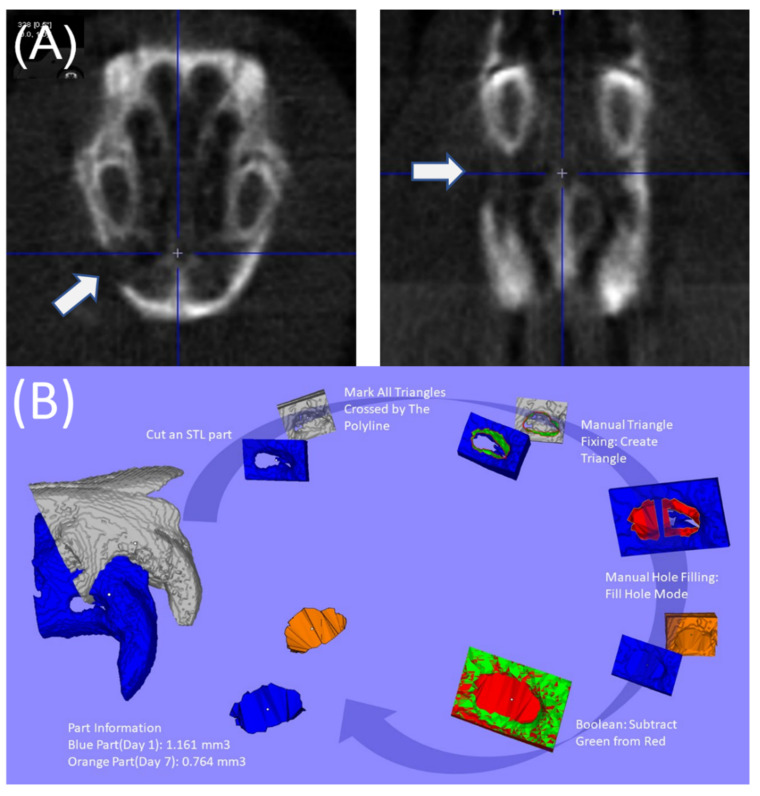
(**A**) Micro-CT scanning of the alveolar bone defect, (**B**) calculation of bone growth in the defect: segmentation of an area involving the defect ➔ incision in stereolithographic (STL) part ➔ marking of all triangles crossed by the polyline ➔ manual triangle fixation to create triangle ➔ manual hole filling with fill hone mode ➔ application of the Boolean method for defect volume.

A NanoSPECT/CT micro-computed tomography device (mCT; Bioscan, Washington DC, USA) was employed to evaluate the evolution of the bone mass and structure of the rats (Figure 3A) at 7 and 14 days post implantation. The mCT images were reconstructed using the Materialize Mimics code (V13.0.1.4, Materialise, Leuven, Belgium), as displayed in Figure 3B.

### 2.10. Statistical Analysis

Paired *t* tests were used to analyze differences between the various groups and were conducted in SPSS software (Version 12.0; SPSS, Chicago, IL, USA). Statistically significant differences were indicated by *p* < 0.05.

## 3. Results

### 3.1. Characterization of Printed Scaffolds and Coelectrospun Nanofibers

**Figure 4:**
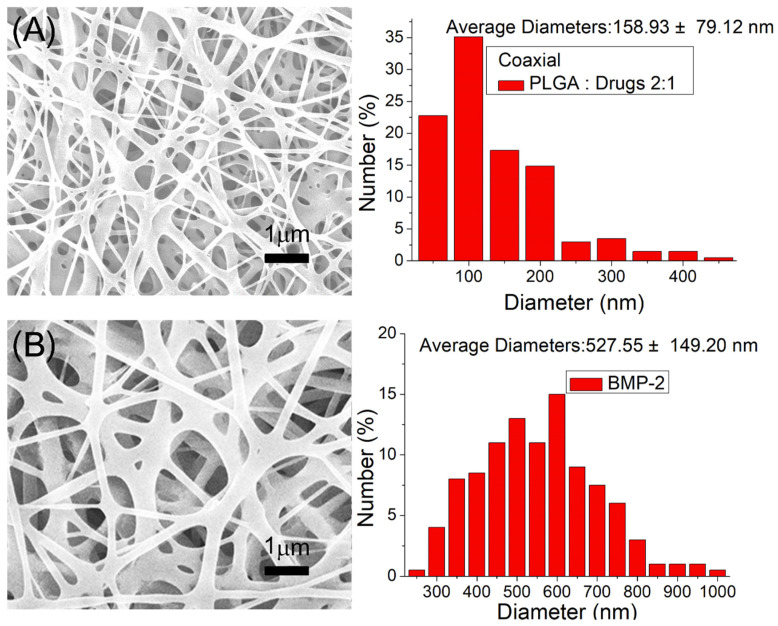
Scanning electron microscopy (SEM) images and fiber diameter distributions: (**A**) drug-loaded nanofibers, and (**B**) BMP-2-loaded nanofibers.

PLA scaffolds were successfully fabricated using 3D printing. Drug- and growth-factor-loaded nanofibrous membranes were also satisfactorily manufactured through the co-electrospinning technique. Figure 4A displays the SEM micro-photos and nanofiber size distributions of ketorolac- and amoxicillin-loaded nanofibers, and Figure 4B does the same for BMP-2-loaded nanofibers. The size of electrospun drug and PLGA nanofibers was (158.9 ± 79.1 nm) smaller than that of the PLGA and BMP-2 nanofibers (527.5 ± 149.2 nm). During the spinning process, the polymer mixture was stretched by the exterior electrostatic force. The inclusion of the drugs in the mixtures reduced the ratio of the polymeric material in the fibers, making the fibers more easily stretched by the exterior electrostatic force, and the diameters of the nanofibers decreased accordingly.

**Figure 5:**
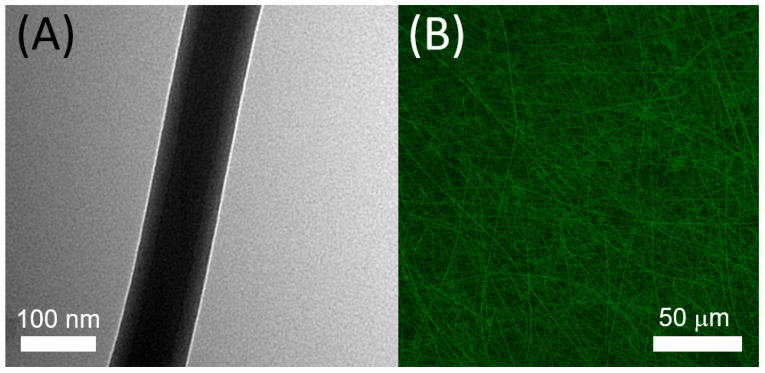
(**A**) TEM of sheath–core-structured nanofiber, (**B**) laser scanning confocal microscopic photo of reGFP in coaxially spun nanofibers.

Figure 5A displays the TEM photo of the coaxially electrospun nanofibers in which the sheath–core structure can be identified. Additionally, Figure 5B illustrates the existence of bioactive proteins in the co-electrospun nanofibers with clearly visible green strings of reGFP, demonstrating that the bioactivity of the biomolecules in the core of the sheath–core-structured nanofibers was successfully maintained.

**Figure 6:**
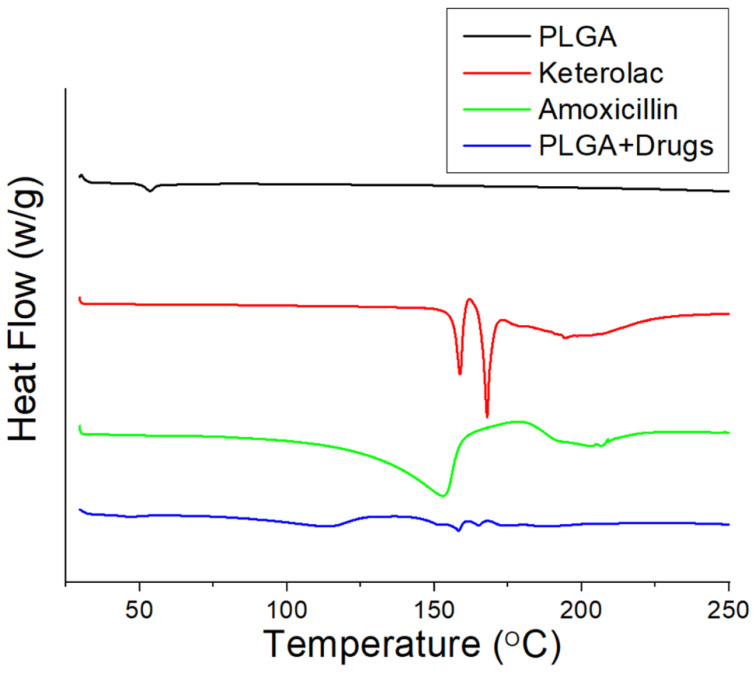
Differential calorimetry scanning (DSC) thermograms of virgin PLGA, ketorolac, amoxicillin, and ketorolac-amoxicillin-incorporated PLGA nanofibers.

Figure 6 illustrates the DSC thermograms of virgin PLGA, ketorolac, amoxicillin, and ketorolac- and amoxicillin-loaded PLGA nanofibers. Although the virgin PLGA exhibited no exothermal calorimetric peaks, various peaks attributable to ketorolac and amoxicillin could be observed in the drug-loaded PLGA nanofibers [34,35], demonstrating that the drugs were successfully incorporated into the PLGA matrix.

**Figure 7:**
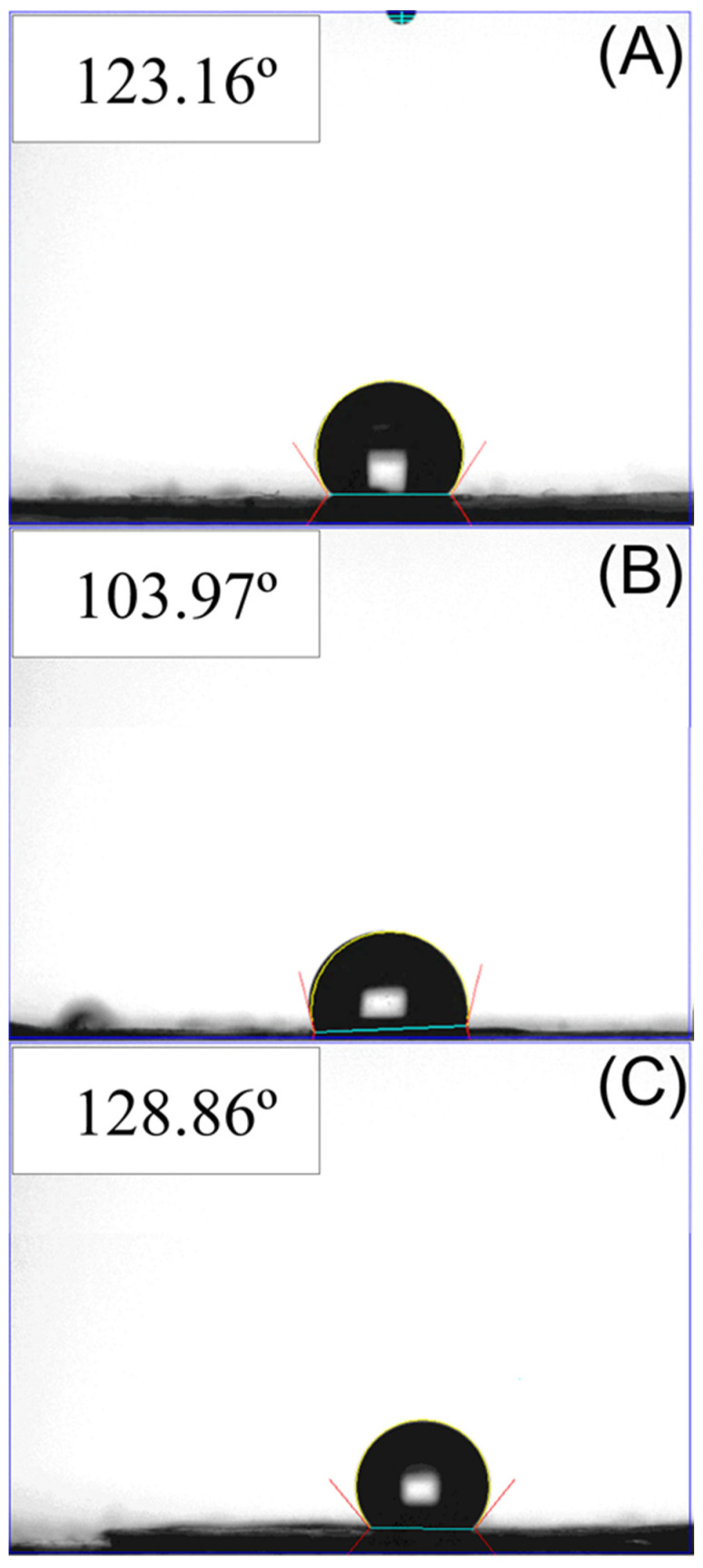
Wetting angles of (**A**) virgin PLGA nanofibers, (**B**) drug-loaded nanofibers, and (**C**) BMP-2-loaded nanofibers.

Figure 7 presents the estimated wetting angles of virgin PLGA, ketorolac- and amoxicillin-loaded PLGA, and BMP-2-loaded PLGA nanofibers. The assessed wetting angle for virgin PLGA nanofibers was 123.2°, and the angles for PLGA loaded with sheath–core-structured ketorolac and amoxicillin, and PLGA nanofibers loaded with BMP-2 were 103.9° and 128.9°, respectively. Although the virgin PLGA and BMP-2-loaded PLGA nanofibers exhibited hydrophobic properties, the inclusion of water-soluble drugs in the PLGA nanofibers moderately increased their hydrophilicity.

### 3.2. Drug and Biomolecule Release Patterns

**Figure 8:**
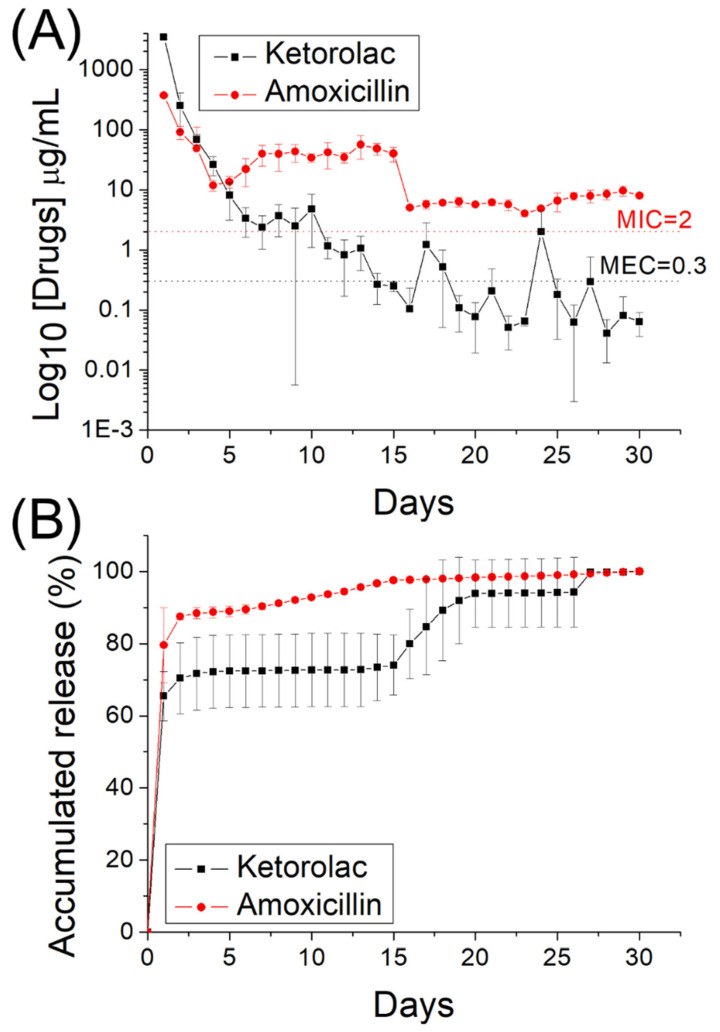
In vitro (**A**) daily and (**B**) cumulative release of ketorolac and amoxicillin from the sheath–core-structured nanofibers. The minimum effective concentration of ketorolac was 0.3 μg/mL, and the minimum inhibitory concentration of amoxicillin was 2 μg/mL [36,37].

**Figure 9:**
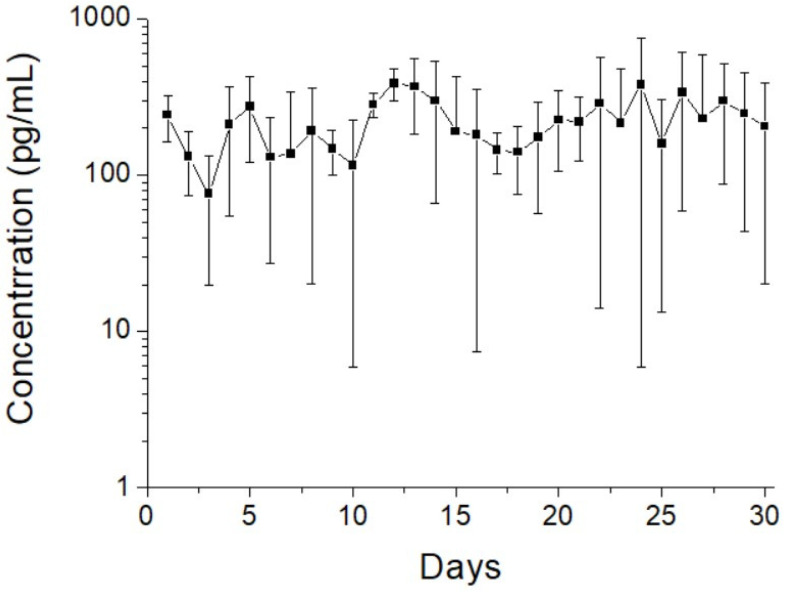
In vitro elution profile of BMP-2 from the sheath–core-structured nanofibers.

Figure 8A,B display the daily and accumulated release patterns of ketorolac and amoxicillin from the nanofibers in vitro, respectively. Ketorolac exhibited a sudden increase on the first day, followed by a slowly decreasing release pattern with minor peaks at days 17 and 23. The release of amoxicillin indicated three-phase discharge behavior, with a primary spike at day 1 day, a secondary peak discharge between days 6 and 14, and a continued steady release thereafter. Due to the sheath–core structure, the nanofibers exhibited sequential-like release. Ketorolac at the sheath layer was released first in a burst during the first 4 days and was then surpassed by amoxicillin beginning at day 5. Additionally, the coaxially electrospun nanofibers provided sustained and effective release of ketorolac (over the minimum effective concentration, MEC) [36] and amoxicillin (over the minimum inhibitory concentration, MIC) for 10 and 30 days, respectively [37]. Additionally, over this period, the ELISA analysis results in Figure 9 indicate that the sheath–core-structured nanofibers provided extended high levels of BMP-2 for >30 days.

**Figure 10:**
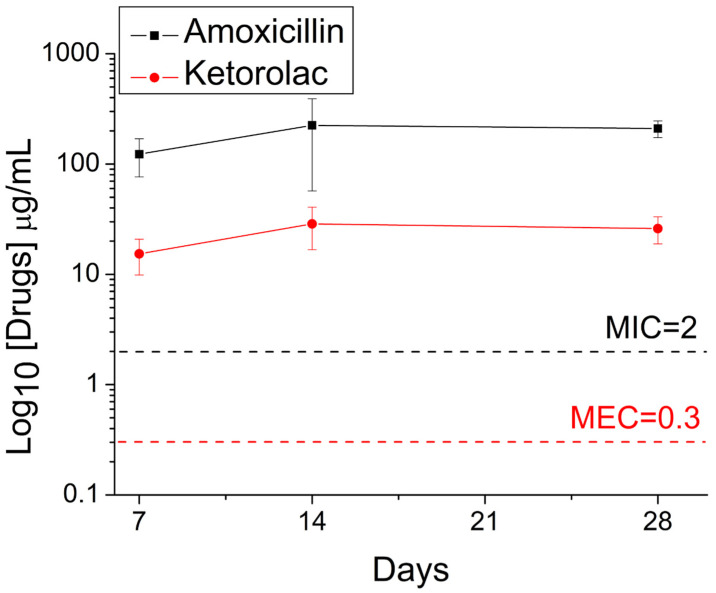
In vivo release of pharmaceuticals from the nanofibers. The minimum effective concentration of ketorolac was 0.3μg/mL, and the minimum inhibitory concentration of amoxicillin was 2 μg/mL [36,37].

The in vivo elution of pharmaceuticals from implanted drug-eluting nanofibrous scaffolds was also investigated. The measured data in Figure 10 suggest that the nanofibers effectively discharged substantial amounts of ketorolac and amoxicillin at the alveolar bone cavity for >28 days in the rats.

### 3.3. Animal Study

**Figure 11:**
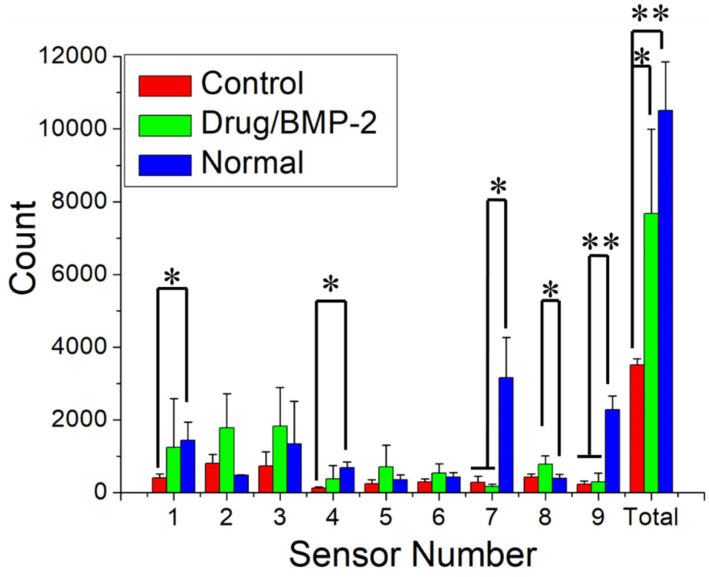
Activity counts when the animals were in a cage for 7 days (* *p* < 0.05; ** *p* < 0.01).

Figure 11 illustrates the sensor counts for the rats in groups over the 7-day postoperative period. The accumulated sensor counts were 3510 ± 173, 7424 ± 480, 7683 ± 2315, and 10,508 ± 1336 for rats in the control (bare scaffold), drug-eluting scaffold, and normal (no surgery) groups, respectively. Rats in the control (bare scaffold) group had significantly lower sensor counts compared with the normal rats (*p* < 0.01). Animals receiving the drug-loaded scaffolds had a significantly greater numbers of sensor triggers than did the animals implanted with the bare scaffolds (*p* < 0.05). Furthermore, the drug and BMP-2 group did not significantly differ with the normal group (*p* > 0.05), demonstrating the efficacy of 3D-printed nanofibrous drug- and BMP-2-loaded scaffolds in the recoveries of the investigated rats.

**Figure 12:**
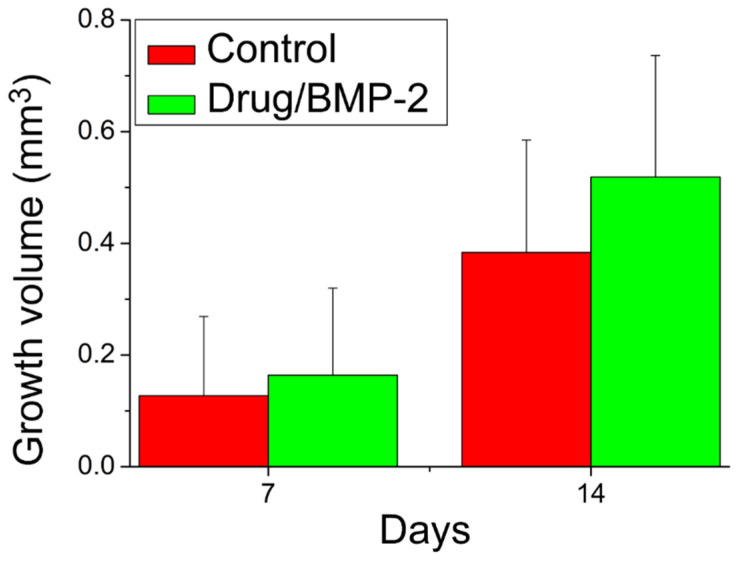
Bone growth in the alveolar bone defect at 7 and 14 days post operation.

**Figure 13:**
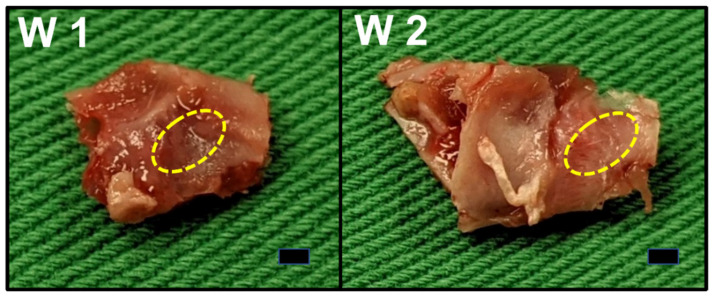
The gross view of bone formation in the cavity (yellow-dotted) at postoperative day 7 (**left**) and day 14 (**right**) (Scale bar = 1 mm).

Figure 12 presents the evaluation results of the bone growth rates based on the mCT images at 7 and 14 days after the implantation of the PLGA scaffolds. Compared with the controls implanted with bare scaffolds, the rats implanted with the drug- and BMP-2-loaded scaffolds exhibited non-significantly (*p* > 0.05) superior bone growth. This non-significance may be because newly grown bones are insufficiently dense to be identified on CT images. The gross images in Figure 13 also indicate that periosteal had formed on the bone surfaces but had not yet reached the consolidation stage where radioactive identification can occur. The experimental results still demonstrated the effectiveness of drug- and BMP-2-loaded scaffolds in accelerating the healing of bone defects.

**Figure 14:**
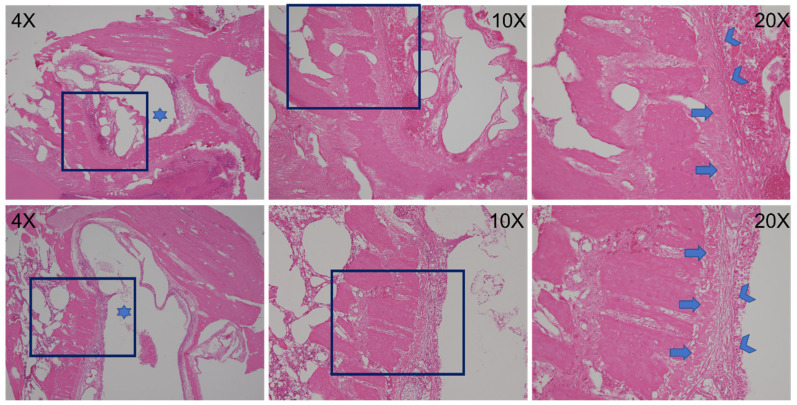
Postoperative day 7 (**upper row**) and day 14 (**lower row**) histology. At the lowest degree of magnification (left, 4× objective), surgically created bone defects were noted (stars). At the highest degree of magnification (right, 20× objective), the bone defects were rimmed by histiocytes (arrowheads, 20× objective). Noted around the bone defect was newly formed woven bone (arrows, 20× objective) that was thicker in the specimen taken on postoperative day 14 compared with that taken on postoperative day 7.

**Figure 15:**
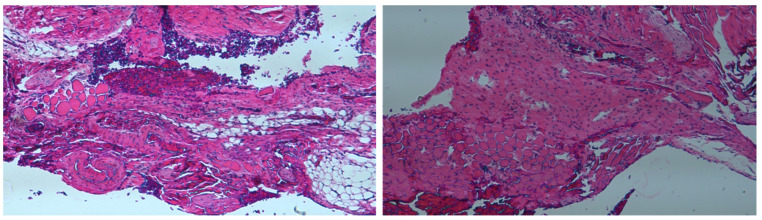
The H&E histological images (magnification: 4.5×) in Control group at day 7 (**left**) and day 14 (**right**). The inflammatory tissue reaction was noted.

Figure 14 displays histological images of bone formation in cavities with scaffold implantation from the BMP group. At a lower resolution (4×), a clear line of bone formation around the border of the cavity could be observed; at a higher resolution (10× and 20×), newly formed osteocytes derived from the bone tissue were observed and the new bone formation can be identified through the presence of layers of histiocytes. In addition, a greater density and maturity of well-aligned osteocytes in the cavity were noted at postoperative day 14 compared with those at day 7. By contrast, the inflammatory tissue reaction was noted in the Control group at day 7 and day 14 post implantation (Figure 15).

## 4. Discussion

Bone defects appear in the alveolar bone of the maxilla and mandible owing to distinct causes, including congenital anomalies, trauma, and osseous deficiency after resection of tumors. Alveolar bone loss results from periodontal disease and successive tooth loss. An autologous bone graft is the preferred method for realigning the alveolar cleft and the maxillary arch to facilitate tooth eruption [1]. Grafts from iliac cancellous bone are currently the most commonly harvested specimens in patients of primary school age. Considering reconstructed 3D images of cavity defects, studies have recommended (1) for 1.5-mL iliac bone grafts to be harvested to meet the demand for defect repair among patients with unilateral alveolar cleft and (2) for 2.5 mL iliac bone graft to be prepared for cases of bilateral alveolar cleft [2]. However, greater amounts of required bone graft equate to greater morbidity at the donor site, as indicated by increases in blood loss, rate of wound infection, and length of hospitalization [3,4]. Therefore, an alternative biomaterial substitute should be developed to mimic the characteristics of iliac bone grafts to enhance bone formation, without the morbidities experienced at the donor harvesting site. The adoption of scaffolds in bone tissue engineering provides an alternative for reforming tissue and regaining its function and aesthetics.

In the present study, we fabricated hybrid degradable PLA scaffolds and drug-eluting PLGA nanofibers for the treatment of alveolar bone defects. The idea of using scaffolds in bone tissue engineering is an essential parameter in the regeneration of critical size bone defects. Scaffolds provide support to cells and growth factors needed for tissue regeneration. In addition, they need to meet the mechanical function of the bone when it regrows [38]. Biomaterials are basically adopted to enhance the organization, differentiation, and proliferation of cells in the procedure of constructing functional tissue by offering structural support, biological containment, and chemical clues [39]. The main function of bone tissues is mechanical support. As a skeletal disorder or tissue damage arises, fixation is needed to restoration the structures included and to establish the appropriate mechanical environment for healing. When healing is realized, the removal of fixation is usually needed. Therefore, degradable polymers are widely adopted in tissue engineering, since they do not require a second operation for removal. PLA is one of the most commonly employed biomaterials due to its easy processability, good mechanical strength and excellent biocompatibility. We therefore selected PLA as the backbone material for the scaffold. In our design of study, the PLA cage with a consistent size 2 mm x 2 mm x 1.4 mm to fit the regular defect was fabricated and embedded adequately into the defect space in the control group. The 3D-printed PLA scaffold could provide the mechanical strength, over which the adhering bone cells would proliferate [40]. One important goal of bone tissue engineering is to realize scaffolds of comparable geometries to those of bone defects. Despite the shape of PLA cage employed to repair the alveolar defects of rats tended to a simple one, scaffolds with implicit surfaces conforming to the geometry of defect cavity can be easily fabricated by using the 3D printing technology. In the experimental group, the PLA cage in combined with the drugs and BMP-2 loaded nanofibers was intended to fully conform to the surrounding bony surface, and sequentially released the drugs/biomolecules to the bony tissues.

Drug release from degradable nanofibers incorporating drugs and biomolecules tends to proceed in three major phases: burst release, diffusion-controlled elution, and degradation-governed discharge. In the electrospinning procedure, a major portion of the incorporated pharmaceuticals are embedded in the main body of the polymeric scaffolding. However, small quantities of the molecules may be distributed on the exterior surfaces of the nanofibers, thus leading to the initial burst release. Subsequently, the discharge behavior is dominated by both drug diffusion and polymeric material degradation. Some deviations were thus noted in the release curves. Minor peaks in drug release were thus noted for amoxicillin between 6 and 14 days, and thereafter, the discharge curve decreased gradually. By contrast, the nanofibers released ketorolac more consistently and continuously. In summary, the measurement results demonstrated that the drug-incorporated nanofibrous scaffolds discharged high concentrations of ketorolac and amoxicillin for 17 and >30 days, respectively. The scaffolds also provided a sustained release of high concentrations of BMP-2. The animal activity analysis indicated that the eluted pharmaceuticals helped to promote the activity levels of the animals in the study, and the mCT images also demonstrated the effectiveness of released BMP-2 in accelerating bone regeneration in the alveolar cavity. These factors are all advantageous for effective pain relief, infection management, and bone regeneration in alveolar defects. In sum, the biomolecule- and drug-incorporated nanofibrous scaffolds demonstrated their effectiveness in the treatment of alveolar cleft.

This study had a few limitations. The first limitation pertains to be the low number of animals enrolled in the in vivo test. Furthermore, our research used a rat alveolar-cleft model to investigate the drug- and BMP-2-incorporated nanofibrous scaffolds. The correlation between the findings in this work and their relevance to analogous alveolar bone defects in humans is unclear and requires additional future investigation.

## 5. Conclusions

Through the use of 3D printing and electrospinning technologies, we successfully developed bioresorbable nanofibrous drug-eluting scaffolds that mimic the structure of the natural EMC of bone tissues. The experimental data suggest that the nanofibrous drug-loaded scaffolds provide an extended release of ketorolac and amoxicillin for >28 days and of BMP-2 for >30 days. The results of in vivo tests also indicated that animals receiving the drug-loaded scaffolds exhibited comparable activity to that of the normal animal group in the study. The histological analysis indicated no signs of adverse effects from the drug-eluting scaffolds. Through the use of 3D printing and coaxial electrospinning, degradable drug- and BMP-2-eluting scaffolds may be successfully manufactured for various maxillofacial applications.
